# Quantitative Estimation of Renal Function with Dynamic Contrast-Enhanced MRI Using a Modified Two-Compartment Model

**DOI:** 10.1371/journal.pone.0105087

**Published:** 2014-08-20

**Authors:** Bin Chen, Yudong Zhang, Xiaojian Song, Xiaoying Wang, Jue Zhang, Jing Fang

**Affiliations:** 1 Academy for Advanced Interdisciplinary Studies, Peking University, Beijing, China; 2 Department of Radiology, Peking University First Hospital, Beijing, China; 3 Department of Electrical Engineering, Chengdu University of Information Technology, Chengdu, Sichuan, China; 4 College of Engineering, Peking University, Beijing, China; University of Florence, Italy

## Abstract

**Objective:**

To establish a simple two-compartment model for glomerular filtration rate (GFR) and renal plasma flow (RPF) estimations by dynamic contrast-enhanced magnetic resonance imaging (DCE-MRI).

**Materials and Methods:**

A total of eight New Zealand white rabbits were included in DCE-MRI. The two-compartment model was modified with the impulse residue function in this study. First, the reliability of GFR measurement of the proposed model was compared with other published models in Monte Carlo simulation at different noise levels. Then, functional parameters were estimated in six healthy rabbits to test the feasibility of the new model. Moreover, in order to investigate its validity of GFR estimation, two rabbits underwent acute ischemia surgical procedure in unilateral kidney before DCE-MRI, and pixel-wise measurements were implemented to detect the cortical GFR alterations between normal and abnormal kidneys.

**Results:**

The lowest variability of GFR and RPF measurements were found in the proposed model in the comparison. Mean GFR was 3.03±1.1 ml/min and mean RPF was 2.64±0.5 ml/g/min in normal animals, which were in good agreement with the published values. Moreover, large GFR decline was found in dysfunction kidneys comparing to the contralateral control group.

**Conclusion:**

Results in our study demonstrate that measurement of renal kinetic parameters based on the proposed model is feasible and it has the ability to discriminate GFR changes in healthy and diseased kidneys.

## Introduction

Glomerular filtration rate (GFR) is the most critical functional parameter of the kidney [1], [2]. Inulin clearance is widely considered the gold standard in quantification of GFR. However, it is not routinely being used in the clinical setting, since it requires multiple blood and urine samples over a period of several hours and could not obtain single-kidney GFR. Traditional radioactive labeled methods are invasive and result in radiation burden. Therefore, it is important to establish a simple new method for quantitative measurements of GFR to monitor renal function.

Recently, on the basis of dynamic contrast-enhanced magnetic resonance imaging (DCE-MRI), functional parameters could be estimated in a wide range of applications including breast imaging [3], brain imaging [4], and abdominal imaging [5]. In order to overcome some mentioned limitations of GFR measurement, several compartment models based on DCE-MRI have been proposed. Patlak-Rutland method was introduced to evaluate glomerular filtration rate, neglecting the outflow of contrast agent from the tubular compartment [6]. Under this assumption, it was often applied to the whole kidney to ensure the tracer stays in the region of interest (ROI) [7], [8], which, however, could not directly reflect GFR since the glomerular filtration predominantly exist in renal cortex [9]–[12].

Subsequently, a two-compartment model with constant dispersion of contrast agent and the consideration of outflow was proposed [13]. However, lacking of accurate tubule parameters estimation was supported and underestimated GFR was found. Later, a similar model was employed to measure GFR in patients by using the whole kidney ROI, which was just simplified by ignoring the dispersion effect in the original two-compartment model [14]. Then, a separable compartment model, of which the time delay of contrast was assumed to be zero [15], provided estimated parameters which were analogues to those of the original two-compartment model [13].

Besides these models, three-compartment models were also proposed for GFR estimations [16], [17]. Nevertheless, the variability of GFR measurement was more pronounced than that of two-compartment models [18]. In addition, the assessment of GFR was sensitive to the segmentation of cortex and medulla, which may result in an unstable measurement [19]. The increase of compartment number would result in an expense of system complexity, and a simple cortical compartment model is alternative because the glomerular filtration occurs in the cortex. We borrowed Zhang et al's idea of using impulse residue function to improve the robustness in GFR measurements [17], and combine it into a two-compartment model.

In this study, a modified two-compartment model was proposed by introducing the impulse residue function to obtain effective estimations of GFR and renal plasma flow (RPF) from DCE-MRI. The advantage of the new model in GFR or RPF measurements over other published models was investigated in Monte Carlo simulation under different noise levels. Then, quantitative estimations were performed in healthy rabbits by using our new model. Furthermore, the proposed model was employed to measure GFR changes in rabbits with unilateral ischaemic acute kidney injury (AKI) to test its validity. Pixel-wise calculation was performed and the cortical GFR results were compared with its contralateral kidney. The major focus of this study was put on the sufficient robustness in kinetic parameters estimation and the ability to discriminate healthy and diseased kidneys.

## Materials and Methods

### Animals

This study was approved by the Ethics Committee on animal care and use of Peking University First Hospital (Ethic number: 10/235). Eight male New Zealand white rabbits (weighing from 2.9 kg to 3.5 kg) underwent this experiment (n = 6 for normal kidney experiment; n = 2 for dysfunction kidney experiment). All the rabbits were housed in individual cages at room temperature, and free fed with standard feed and tap water, and stopped 12 hours before experiments. Before DCE-MRI, pentobarbital sodium (0.5 ml/kg body mass) was injected through the marginal ear vein with a 24-gauge catheter for anesthetization. All the animals were placed in a supine position in a fixed device to limit abdominal motion during scans. Heart rate was continuously monitored by using a photopulse sensor of the MR scanner during acquisitions.

In the experiment of dysfunction kidneys, two rabbits (2.9 kg and 3.3 kg) were implemented a unilateral renal ligation surgery procedure after anesthetization. After a midline incision, the aorta of left kidney was clamped for 45 minutes to induce ischaemic acute kidney injury. The right kidney was normal and regarded as the control group. After surgery, we sew up the incision with sewing wires and give analgesic treatment to minimize suffering. The animals' body temperature was maintained about 38° by using a hotplate during surgery and exposed to an infrared light in the box before MR scans. Two days later, the DCE-MRI experiment was implemented. These two injured rabbits (acute ischemia in the left kidney) were scanned together with all the other six normal rabbits.

### MRI

The abdomen dynamic images were acquired on a whole body 3.0 T MR scanner (Signa Excite; GE Medical Systems, Milwaukee, WI, USA) with TORSOPA coil. Before each DCE-MRI scan, T1 measurement was conducted firstly with a three-dimensional spoiled gradient-recalled echo sequence. T1 mapping was measured by using variable flip angles method [20], and the imaging parameters are as follows: TR = 6.2 msec, TE = 2.9 msec, flip angle = [3], [9], [20]°, slices = 16, slice thickness = 4.0 mm, matrix = 256×256, FOV = 200×200 mm^2^. Then, DCE-MRI scans were performed by a three-dimensional fast spoiled gradient recalled echo sequence with the following parameters: TR = 3.3 msec, TE = 1.3 msec, flip angle = 15°, FOV = 160×160 mm^2^, ASSET = 2, acquisition matrix was 128×128 and interpolated to 256×256, slice thickness = 4.0 mm, slice number = 16, bandwidth was 488 Hz/pixel, and acquisition time was 3.0 s/frame. Five precontrast frames were obtained before bolus injection, then, an administration of 0.05 mmol/kg of Gd-DTPA (Omniscan; GE Healthcare Ireland, IDA Business Park, Carrigtohill, Co.Cork) was performed with a venous cannula at a rate of 2 ml/s. 5 ml of saline was immediately flushed in and totally 100 frames were obtained in about five minutes.

### Data Analysis

Renal parenchyma and the aorta were automatically segmented from the surrounding tissue with a Level-Set framework [21]. The parameters used in segmentation were: alpha = 0.04, a parameter that controls the weight of smoothing item of the image features; and iteration was set to 60, such values were sufficient for detection of kidney outline in this experiment. Afterwards, dynamic images of the segmented regions were registrated to reduce motion [22]. Then, a slice which covering the largest possible parenchyma during corticomedullary phase was used for cortical ROI drawing to obtain the tissue signal intensity curves. The size of ROIs for all the kidneys was 590 pixels in average. The arterial input function (AIF) was determined by drawing ROI in the slice that a branch of renal artery was obviously seen, that is, the ROI was placed within the aorta distal to the branch of the renal artery. To improve the robustness of AIF and reduce the inflow artifacts, one more slice above was also selected for averaging. A 3×3 pixel size ROI was used. Furthermore, the tail of the AIF was fit to a biexponential decay to reduce respiratory motion related noise [23].

Then, signal intensity curves were converted into gadolinium concentrations with the T1 values from T1 mapping [23], under the assumption that a linear relationship between relaxation rate changes and [Gd] concentrations according to the following equation:
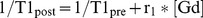
(1)where, 1/T1_pre_ and 1/T1_post_ are the contrast relaxation rates before and after bolus injection. r_1_ is the specific T1 relaxivity at physiological temperatures in plasma (4.1 L s^−1^ mmole^−1^) [24], [25].

### The New Compartment Model

**Figure 1:**
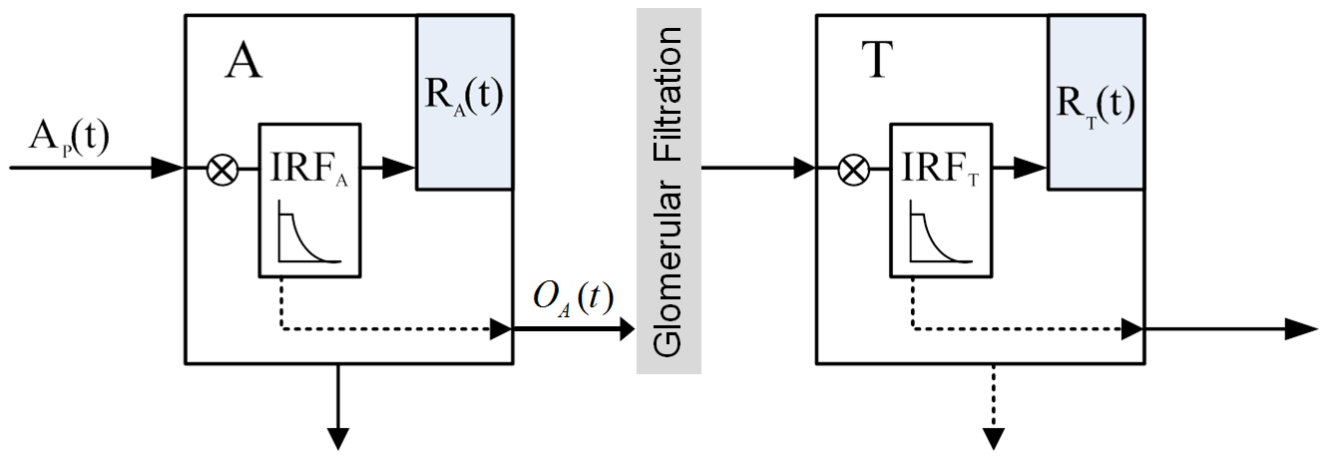
Schematic diagram of the modified two-compartment model with impulse residue function for glomerular filtration. A is the vascular compartment includes intrarenal arteries and glomerular vessels, and T is the tubules compartment. The retention function R_A_ and R_T_ in compartment A and T (represented in solid arrow within the box) are the convolution of the input and each impulse residue function. Dashed lines denote the outflow of each compartment and the outflow of compartment A partially flows into compartment T.

The proposed model in this study describes two compartments: the intrarenal arteries and glomerular vessels (A) and the renal tubules in cortex (T), with tracer flowing from compartment A into compartment T, shown in Fig. 1. The concentration in descending aorta is expressed as the arterial input A_0_(t), and the concentration in cortex, C_cortex_(t) is contributed by the retention in compartment A and T over time. The arterial input A_0_(t) is converted into plasma concentration A_p_(t) by dividing by (1-Hct), where Hct = 0.45 is the hematocrit for rabbits.

While contrast passes through the kidney, an administration of impulse residue function (IRF) can be useful to describe the characteristics of contrast agent distribution in each compartment [26]. In our study, considering the dispersion effect and transit time of contrast, piecewise-exponential impulse residue function is introduced to determine its response to the idealized bolus injection. For an ideal instantaneous unit bolus injection, it is actually a time-enhancement function [27]. The contrast is considered to be a bolus injection into the kidney and equals 1 (t<τ_i_), then an exponential fall with constant rate m_i_ starts when the contrast is washing out (t>τ_i_), and the parameter τ_i_ is the minimal transit time:
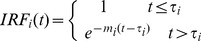
(2)where the 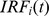 is the impulse residue function and i represents A and T.

With the proposed model (Fig. 1), the residue amount of contrast in each compartment after a bolus injection is presented as such a convolution of the input and its corresponding impulse residue function:
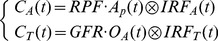
(3)where 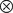 express the convolution, RPF is the renal plasma flow, GFR is the glomerular filtration rate, and C_i_(t) is the contrast concentration in each compartment.

In renal system, we defined the retention function R_A_ and R_T_ to illustrate the changes of contrast for each compartment after the convolution of IRF_i_. We assumed the unit input for compartment A is 1, then the retention function of A equals IRF_A_(t), however, the unit input changes in compartment T. Because the input for T is from the output of compartment A, the retention function for T is the convolution of O_A_(t) and IRF_T_(t), then equation 3 becomes:
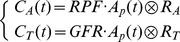
(4)where
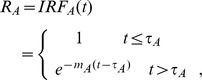





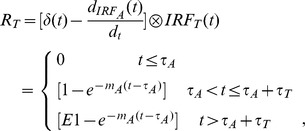




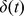
 is the unit input and then cortical retention function written as: 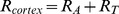.

The mean transit time (MTT) was defined as the area under the retention curves according to the equation: 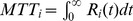.

Thus, the amount of contrast remained in cortex region is given by: 
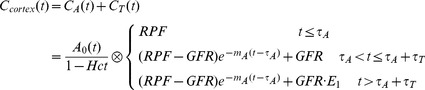
(5)where 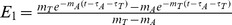, C_cortex_(t) is the concentration in cortex. Parameters were fitted by using the nonlinear least-squares algorithm.

### Monte Carlo Simulation

To evaluate the reliability of the new model compared with other published models in estimating GFR in rabbit kidney, Monte Carlo simulations were conducted. Artificial aortic input function used in this simulation was obtained by smoothing an AIF curve with temporal resolution of 3 second. Smoothing was carried out by a gamma variante function [28].

Totally six compartmental models were included with the same preset GFR values in this simulation: Patlak-Rutland method (GFR = 3.0 ml/min, v_a_ = 2.4), PR [6]; a two- compartment model (GFR = 3.0 ml/min, k_out_ = 0.08, f_a_ = 0.2, d = 0.42), 2CD [13]; a simplified two-compartment model (GFR = 3.0 ml/min, k_out_ = 0.08, f_a_ = 0.2), 2C [14]; a separable compartment model (GFR = 3.0 ml/min, T_t_ = 1/k_out_  = 12.5, V_p_ =  f_a_  = 0.2, T_p_ =  d  = 0.42), SP [15]; a three-compartment model (GFR = 3.0 ml/min, RPF = 3.0 ml/g/min, f_p_ = 0.18, w_a_c_ = 0.2, w_a_m_ = 0.08, w_p_ = 0.18), 3C [16]; and the proposed two compartment model with impulse residue function in this study(GFR = 3.0 ml/min, RPF = 3.0 ml/g/min, m_A_ = 0.2, τ_A_ = 3.2 s, m_T_ = 0.1) and τ_T_ was assumed to be 0, 2C-IRF. Then, the tissue curves were generated from their corresponding initial parameters and the artificial AIF. Considering the impact of noise in DCE-MRI scans, different levels of noise were reintroduced to each concentration curve in Monte Carlo simulation. Guassian noise with zero mean and SD equals to 2%, 3%, 5%, 10%, and 15% of the mean magnitude of each gadolinium concentration curve were used. These noise were generated by using randn(1,N) function in Matlab and be added to construct noisy tissue curves. All these models were used to fit its corresponding noisy data for which true values of these parameters were known. 2000 Monte Carlo trials were conducted, and the variability of each fitted parameter was obtained. In order to assess the variability of the estimated parameter, the coefficient of variation (CV) was used according to the equation: CV = SD/mean. The bias in estimated parameters can be given by the difference between mean value of 2000 simulations and the actual values.

### In-vivo Experiment

After testing the reliability and bias of the proposed model in parameters estimation and comparing the precision of GFR measurement with other published models, the extracted concentration curves from DCE-MRI data were used for the measurements of kinetic parameters with 2C-IRF model in normal rabbit kidneys. Nonlinear least squares fitting was implemented with all the data sets by using Levenberg-Marquardt algorithm. To assess the goodness of fit, coefficient of determination, denoted R^2^, was also calculated.

Acute kidney injury causes significant renal dysfunction in terms of decreased glomerular filtration rate [29]. In order to investigate the validity of the proposed model to detect GFR alterations in such an abnormal condition, pixel-wise calculation of GFR were measured with the dysfunction kidney, as well as the contralateral kidney. To further quantify the cortical mean GFR in the abnormal kidneys, two-certified radiologists with at least 5 years of experience in renal DCE MR imaging, who were blinded to the experiment, draw the cortical ROI on the T1-weighted images and the mean GFR values from its corresponding mapping image were obtained.

### Histology

Kidneys in dysfunction experiment were fixed in 10% neutral buffered formalin and embedded in paraffin for light microscopic study. Kidneys were sectioned into 3-µm slides and stained for histology with hematoxylin-eosin. One experienced pathologist, who was blind to which experimental group the samples belonged, reviewed histological findings.

### Statistical Analysis

Results of the quantitative measurements are expressed as mean 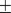 SD. Paired t-test was performed and a P value<0.05 was considered statistically significant. Coefficient of determination (R^2^) is calculated to assess the goodness of fit. All analysis are implemented in Matlab (MathWorks, Natick, MA).

## Results

### Monte Carlo Simulation

**Figure 2:**
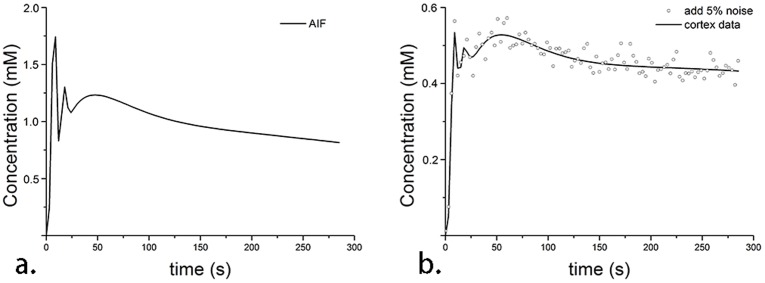
Concentration curves in Monte Carlo simulation for a.) the artificial AIF and b.) the tissue curve which is generated with the known initial parameters and added with 5% level noise.

In Monte Carlo simulation, the artificial AIF is shown in Fig. 2a, and 2%, 3%, 5%, 10% and 15% noise were reintroduced into the concentration curves at all the time points, shown in Fig. 2b with 5% noise for instance.

**Figure 3:**
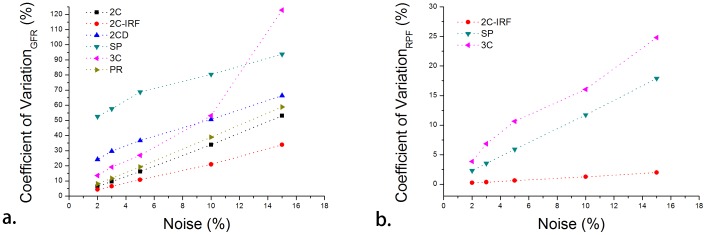
Monte Carlo simulation results for each compartment model in estimating a.) GFR and b.) RPF, only three models could extract RPF. Lowest variability of estimated GFR and RPF (red dotted line) are found in the proposed two-compartment model at 2%, 3%, 5%, 10% and 15% noise, respectively.

Compared with other five published models, the smallest CV values of estimated GFR were found in 2C-IRF model, seen in Fig. 3a. Higher variability was found in the other compartment models in GFR estimate and pronounced impact existed in 3C model when noise level was above 5%. The bias of estimated GFR was 0.01%–4.0% for 2C-IRF, 0.2–11.3% for 2C, 2.6%–9.8% for 2CD, 8.4%–14.7% for SP, 1.1%–17.1% for 3C, and 0.2%–1.7% for PR. For RPF Monte Carlo simulation, smallest CV values were also found in 2C-IRF model, ranging from 0.2% to 2.0%, seen in Fig. 3b. The other models have a bigger variability of more than 10% when the noise level is above 5%. The bias of estimated RPF of 2C-IRF model was 0.05% to 0.3%, much lower than those of other models.

The estimated GFR and RPF results are robust under noise conditions by using 2C-IRF model. Small variability of estimated RPF was found with CV values ranging from 0.2% to 2.0%, while CV values of GFR were 4.2% to 10.8% under 10% noise. The bias of the estimated GFR, RPF from the new model was low with a range of 0.0%–0.6% for different noise levels, excluding one larger bias of 4.0% for GFR at 15% noise level.

### In-vivo Experiment

**Figure 4:**
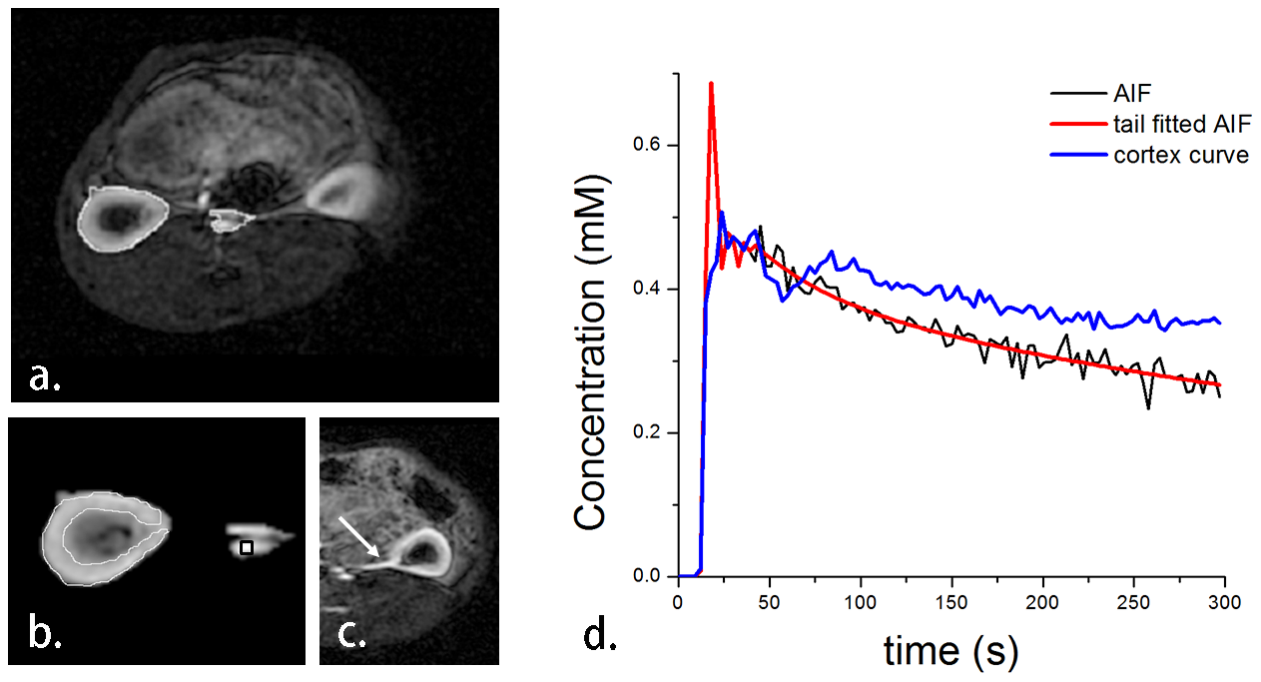
A representative image from DCE-MRI acquisition. a.) showing the region of interest after segmentation; b.) the manually drawn cortical ROI (white line); c.) the image in which a branch of renal artery is clearly seen is used for aortic ROI drawing; and d.) the concentration curves of each corresponding ROI. The original aortic input function (black line) fits its tail with a biexponential method (shown in red line). The blue line is the cortical concentration curve.

In this study, all the rabbits were analyzed with the 2C-IRF model for kinetic parameter estimations. A segmentation result is shown in Fig. 4a. After registration, cortical ROI was manually drawn (shown in Fig. 4b) and slice that with branch of renal artery (white arrow in Fig. 4c) was selected for aortic ROI drawing. Representative concentration curves derived from corresponding ROIs are shown in Fig. 4d.

**Figure 5:**
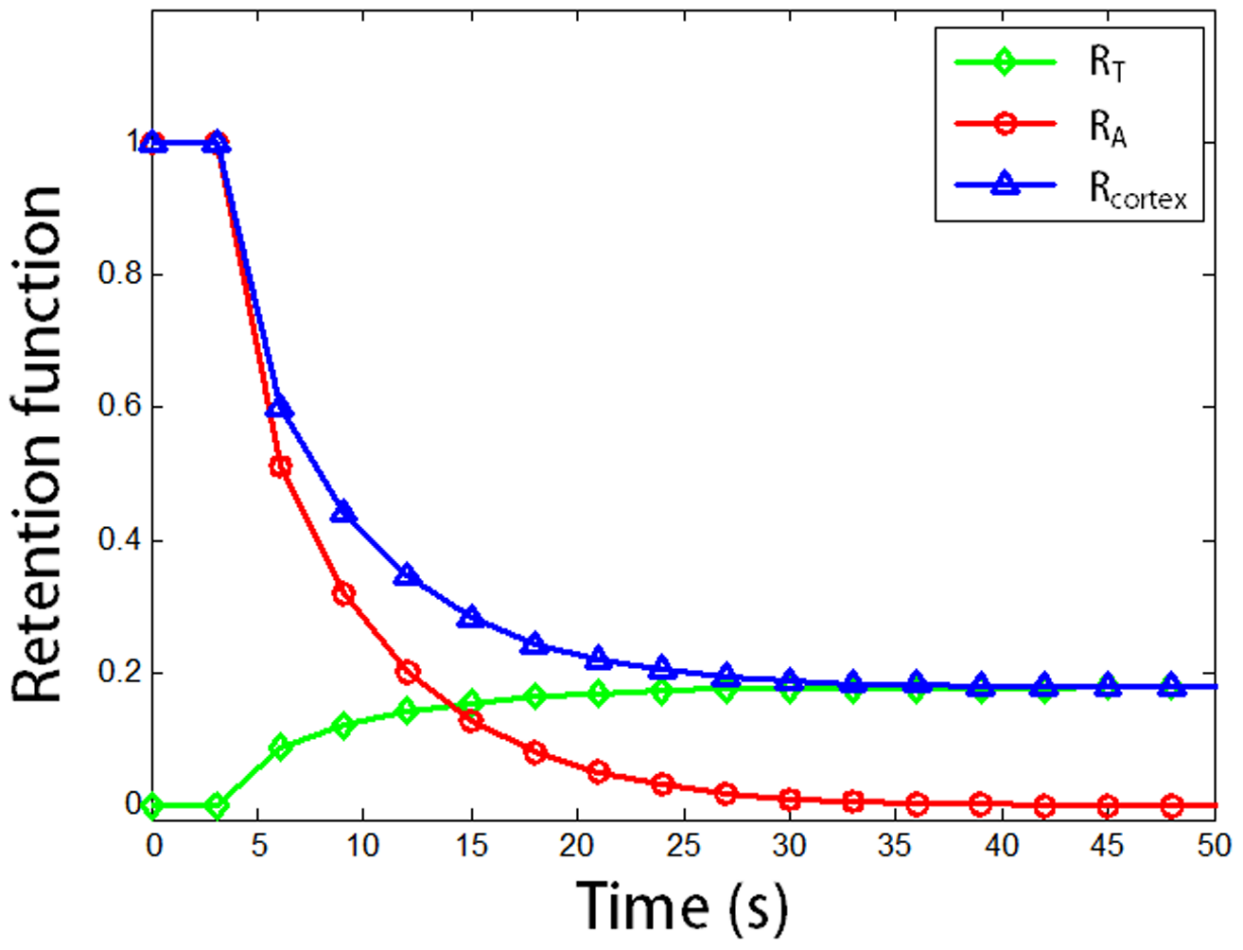
Typical retention function curves for vascular compartment (R_A_, red), tubule compartment (R_T_, green) and the cortex region (R_cortex_, blue).

Estimation results of all the kidneys are listed in Table 1. GFR obtained from the new model was 3.03±1.1 ml/min, RPF was 2.64±0.5 ml/g/min, vascular mean transit time MTT_A_ was 5.6±0.6 s, tubule mean transit time MTT_T_ was 15.1±8.8 s and the mean transit time of the kidney MTT_K_ was 20.7±8.7 s in average. No significant differences were found for GFR, RPF, MTT_A_, MTT_T_ or MTT_K_ when comparing the left and right kidney groups (p>0.05 for all). Satisfactory goodness of fit was found for all the cases, with R^2^ ranged from 0.81 to 0.97. Typical retention curves are shown in Fig. 5.

**Table 1:** Estimated parameters of normal kidneys using the proposed two-compartment model and goodness of fit for the nonlinear fitting.

Num.	GFR	RPF	τ_A_	MTT_A_	MTT_T_	MTT_K_	R^2^
	(ml/min)	(ml/g/min)	(s)	(s)	(s)	(s)	
1_L	3.25	2.85	2.96	5.51	13.15	18.66	0.95
2_L	3.44	2.65	2.76	4.95	25.47	30.42	0.88
3_L	3.85	2.15	1.85	5.91	20.81	26.72	0.97
4_L	2.92	3.71	3.77	6.52	10.07	16.59	0.94
5_L	1.08	2.07	1.85	4.80	6.35	11.15	0.95
6_L	2.42	3.12	1.06	5.62	3.95	9.57	0.81
mean	2.83	2.76	2.37	5.55	13.3	18.85	0.92
(±SD)	0.98	0.61	0.97	0.63	8.37	8.32	0.06
1_R	3.58	2.55	2.61	5.20	16.15	21.35	0.94
2_R	3.69	2.40	2.48	4.70	30.66	35.36	0.83
3_R	3.77	2.10	1.67	6.40	23.35	29.75	0.97
4_R	4.87	3.04	3.19	5.92	19.42	25.34	0.94
5_R	0.94	2.19	1.98	5.17	5.36	10.53	0.96
6_R	2.59	2.85	1.20	6.36	6.90	13.26	0.82
mean	3.24	2.52	2.19	5.63	16.97	22.60	0.91
(±SD)	1.33	0.37	0.71	0.70	9.70	9.55	0.06

Note: L represents the left kidney and R represents the right kidney in normal rabbits.

**Figure 6:**
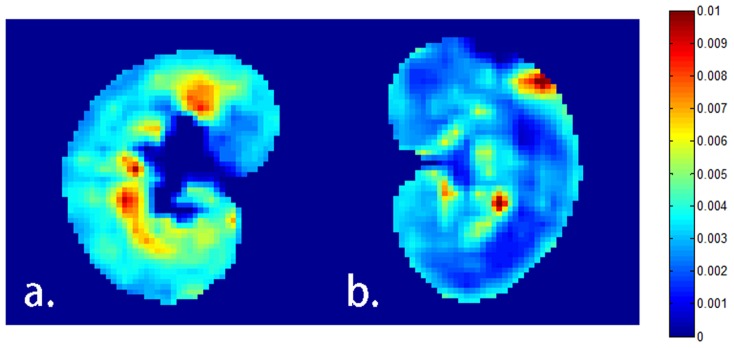
GFR mapping of a.) the normal kidney (right kidney) and b.) the acute ischemia kidney (left kidney) by using the new model. The left kidney of the rabbit is under surgical ligation for totally 45 minutes, and lower GFR values are clearly observed in the cortex and outer stripes of the outer medulla regions while corresponding regions are high in the control group.

**Figure 7:**
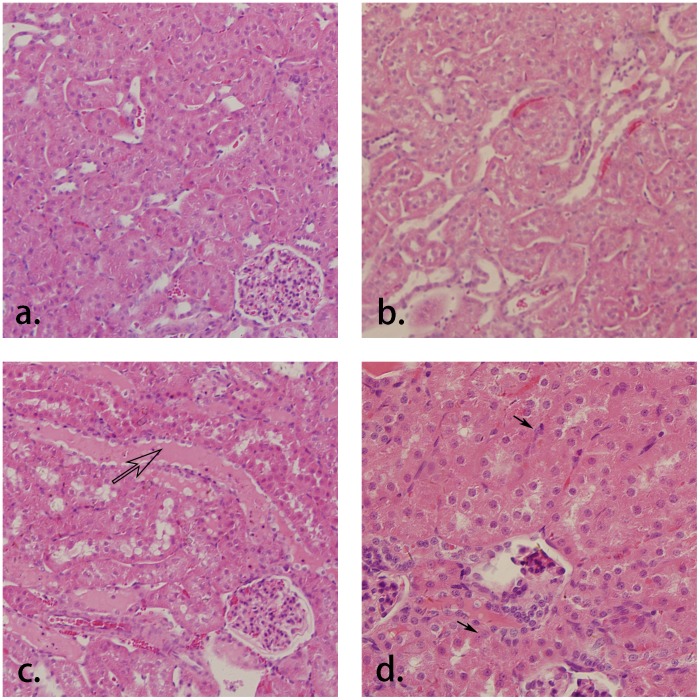
Histology sections of a.) the normal kidney (H&E, ×100) and the acute ischemia kidney with histological findings of b.) tubular dilatation (×100), c.) cast deposition (open arrow, ×100) and d.) cell necrosis (black arrow, ×200).

In dysfunction kidney experiments, large decrease of GFR was found in the cortical region of the left kidneys, shown in Fig. 6b, while the right kidneys (control group, Fig. 6a) remain normal. Pixel-wise calculation was implemented and the GFR mapping shows the differences between the dysfunction kidney and the contralateral normal kidney, which are in good concordance with the cortical mean values that listed in Table 2. The changes of the kidney with acute injury are particularly visible on histology compared to the normal kidney (Fig. 7a). Histological findings show main tubular dilatation (Fig. 7b), cast deposition (open arrow, Fig. 7c) and cell necrosis (black arrow, Fig.7d) in the injured kidney.

**Table 2:** Pixel-wise estimation of GFR for dysfunction kidneys by using the proposed two-compartment model.

	Cortical	Region
Kidneys	pixel GFR	mean GFR
	(ml/min)	(ml/min)
Injury 1.	0.0037	2.27^a^
Control 1.	0.0080	4.55
Injury 2.	0.0042	2.78^a^
Control 2.	0.0068	4.73

a pronounced reduction was found compared to the control group.

## Discussion

In present study, a modified two-compartment model with impulse residue function is implemented, and Monte Carlo simulation results indicate the reliability of the proposed model in parameters estimates. The main result of this study is that the GFR and RPF measurements are in close agreement with literature results in rabbits. Moreover, the new model is valid in detecting GFR alterations in diseased kidneys. The GFR value of normal kidneys in our study is 3.03±1.1 ml/min (n = 6), which is comparable to a mean value of 2.9±1.0 ml/min measured in rabbits using a deconvolution method [30] and model derived GFR of 2.3±1.0 ml/min with original two-compartment model and 2.8±1.2 ml/min with Patlak-Rutland method reported in rabbits study (n = 10) [13]. Here, GFR results of each compartment model for in-vivo experiments are presented in Table 3. The close agreement with literature results for the proposed model is found.

**Table 3:** Estimated results of GFR for each two-compartmental model.

n = 6	2C-IRF	2CD	2C	SP	PR
GFR(ml/min)	3.03	1.29	1.55	1.80	0.84
(±SD)	1.1	0.7	1.1	0.6	0.5

Moreover, the RPF measured in this study is 2.64±0.5 ml/g/min, which is in close agreement with literature reports [22], [30]–[33], shown in Table 4. Our results resemble the renal perfusion reported in rabbits and larger than the deconvolution method (Hermoye L et al.). The perfusion results in rats (Zimmer F et al., 4.16±1.24 ml/g/min based on arterial spin labeling method and 5.42±0.85 ml/g/min based on DCE method) are larger than the results in our study (2.64±0.5 ml/g/min), this discrepancy underscores physiological differences across species. The close agreement with literature and the valid assessments of discriminating healthy and dysfunction kidneys indicate that the proposed 2C-IRF model provides a useful method for quantitative measurements in kidneys, and the difference in accuracy is relevant. Our results show that the new model is feasible and not only achieves reliable measurements of renal function, but also be capable in detecting of GFR alterations in dysfunction kidneys.

**Table 4:** RPF values in literature studies.

Authors	year	ml/g/min	method
Ott CE et al. [31]	1979	3.2±0.3 (n = 13)	inulin clearance
Hermoye L et al.* ^a^ [30]	2004	1.3±0.4 (n = 6)	deconvolution
Winter JD et al. [22]	2011	3.28±0.59 (n = 5)	ASL* ^c^ based
		2.98±0.60 (n = 6)	DCE* ^d^ based
Zhang Y et al. [32]	2012	3.20±0.67 (n = 9)	ASL based
Zimmer F et al.* ^b^ [33]	2013	4.16±1.24 (n = 6)	ASL based
		5.42±0.85 (n = 6)	DCE based

Unit is ml/g/min.

*^a^unit converted to ml/g/min with 1 g/ml density.

*^b^rat models were used in RPF measurements. Five of the six rats had a unilateral ischaemic AKI and total seven healthy kidneys were included in the calculation of mean RPF.

*^c^arterial spin labeling (ASL) MRI.

*^d^dynamic contrast-enhanced (DCE) MRI.

Using the predefined impulse residue function, which considers the transit time and dispersion effect of contrast agent in kidney, our new model could characterize the distribution of contrast and elucidate physiological meaning better, as well as improves the model reliability with reduced bias in measurements of parameters. A similar use of impulse residue function approach has been reported [17]. However, in order to increase the accuracy of the estimated parameters, they introduced more compartments, which results in the expense of system complexity.

In this study, the smallest variability of estimated GFR and RPF is found in 2C-IRF model, which illustrates that it is not sensitive to noise and sufficiently robust in GFR or RPF measurements from DCE-MRI. Usually, deconvolution method is clinically used to determine renal transit time, however, it continues to exhibit noise in the deconvolved curves. By using the impulse residue function in our model, the vascular mean transit time is 5.6±0.6 s, which is similar to the result (6.8±1.7 s) in a previous study [30], and we also generated the tubule mean transit time (15.1±8.8 s) and kidney mean transit time (20.7±8.7 s).

In DCE-MRI scans, administration of gadolinium contrast is usually used to enhance the signal. Gd contrast agents are rapidly cleared with a half-life of about 2 h in normal kidneys, however, it would exceed in patient with dysfunction kidneys. Thus, the retained and subsequent retention of Gd contrast would activate illness known as the nephrogenic systemic fibrosis (NSF) disease [34], [35]. Researchers demonstrated Gd contrast possibly plays the triggering role in the development of NSF [36]. Thus, higher dose of Gd contrast would more easily lead to NSF disease. In our study, low dose of contrast about 0.05 mmol/kg was used and this may reduce the risk of NSF.

There are several limitations in our study. First, the population of the dysfunction kidneys is small. Second, the arterial input function may be affected by the inflow effects in the aorta. Last, the validity of the new model should be tested in human kidneys before clinical utility.

In conclusion, our new model with the introduction of impulse residue function is feasible and suitable to estimate important renal functional parameters, and has the ability to discriminate GFR changes in healthy and diseased kidneys.

## Supporting Information

Checklist S1 **ARRIVE Checklist.**
(DOC)
